# Neurological Spectrum of Epstein-Barr Virus Infection: A Rare Clinical Case

**DOI:** 10.7759/cureus.53302

**Published:** 2024-01-31

**Authors:** Dora Gomes, Rui André, Catarina Oliveira, Sofia Camões, Catarina R Silva, Miguel L Mendes

**Affiliations:** 1 Internal Medicine, Centro Hospitalar Tondela-Viseu, Viseu, PRT; 2 Neurology, Centro Hospitalar Tondela-Viseu, Viseu, PRT

**Keywords:** nflammatory response, central nervous system infection, intravenous immunoglobulin, polyradiculoneuropathy, epstein-barr virus

## Abstract

The Epstein-Barr virus (EBV) is a DNA virus that has been infecting humans since ancient times, capable of causing a wide range of pathologies and affecting approximately 90% of the population.

A 61-year-old male with no significant medical history presented with a 5-day history of imbalance and difficulty walking. Neurological examination revealed specific findings, including absent reflexes, bilateral asynergy, and gait abnormalities. Contrasting with Guillain-Barré Syndrome, lumbar puncture suggested a central nervous system infection. Serological testing confirmed Epstein-Barr virus (EBV) positivity, and intravenous immunoglobulin led to significant improvement. Electromyogram results suggested inflammatory/ipnfectious polyradiculopathy. Repeat EBV serology, showing strongly positive IgG and negative IgM, confirmed the diagnosis of Polyradiculoneuropathy secondary to EBV. This case underscores the rare neurological complications of EBV and the importance of considering viral infections in such presentations.

## Introduction

The Epstein-Barr virus (EBV) is a DNA virus present in humans since ancient times, capable of causing a wide clinical spectrum [1]. Complications involving the nervous system due to EBV are rare, occurring in 0.5 to 7.5% of patients [2]. However, they can affect both the central and peripheral nervous systems, leading to conditions such as aseptic meningitis, encephalitis, and mono- and polyradiculitis.

Although the precise pathophysiology of the involvement of the nervous system by EBV is not fully understood, it is believed that the direct effects of EBV on the microvasculature and the secretion of inflammatory cytokines released during B cell infection may be involved [1]. This clinical case highlights the existence of rare neurological manifestations of a viral infection that affects approximately 90% of the population.

## Case presentation

A 61-year-old Caucasian man, previously healthy and an active smoker (approximately 21 pack-years), who works as a teacher, presented to the emergency department with a complaint of imbalance and difficulty in walking, predominantly in the morning. He reported experiencing dysesthesia in the knees during the nighttime, with a progression of symptoms over the past 5 days. The described symptoms did not show any changes, and the patient denied ascending paresthesias, alterations in sphincter function, dysphagia, shortness of breath, cranial nerve deficits, dizziness, headaches, visual or language changes, and loss of consciousness. No fever was reported, and there were no recent travels or relevant epidemiological context. The patient also mentioned symptoms of rhinorrhea and a non-productive cough with a duration of 1 week.

The patient had previously been evaluated elsewhere, with laboratory results revealing 17% atypical lymphocytes, GGT 1862 IU/L, ALT 219 IU/L, alkaline phosphatase 636 IU/L, and a total bilirubin of 3 mg/dL, primarily due to direct bilirubin (2.33 mg/dL). A CT angiogram showed no signs of acute vascular injury, and an abdominal ultrasound revealed lymph node formation in the hepatic hilum measuring 27x11 mm.

Upon objective examination in the emergency department, the patient was hemodynamically, afebrile, breathing comfortably in room air, with a healthy complexion, well-hydrated, non-cyanotic, and non-jaundiced. No abnormalities were noted during cardiac and pulmonary auscultation. Abdominal palpation revealed a soft, depressible abdomen without tenderness or apparent masses/organomegaly. Examination of the oral cavity showed erythema without apparent exudates, and no apparent cutaneous changes were observed.

Neurological examination revealed the patient to be alert, conscious, self and externally oriented in time and space, and cooperative with the clinical history. There were no signs of dysphasia, dysarthria, or dysphonia. No meningeal signs or neck stiffness were present. The eyes were orthophoric, with isocoric, reactive, and symmetric pupils. Oculocephalic movements were unrestricted, and there was no nystagmus. No sensory alterations were noted in the trigeminal nerve. No facial weakness was observed. The tongue was in the midline. The patient exhibited a very discreet widening of the base during gait, was unable to perform tandem walking, and had difficulty with heel-to-toe walking. Superficial painful sensitivity was normal, except for irregular lateral areas of hypoesthesia in the lower limbs, not corresponding to anatomical areas. Vibration sensitivity was 8 to 10 seconds at the internal tibial malleoli. No abnormal involuntary movements were present. No obvious motor lateralization was noted in the arms outstretched test, Mingazzini maneuver, or segmental strength tests. There were no alterations in axial coordination or appendicular kinetics in the finger-nose or heel-knee tests, except for some hesitation/asynergy in the latter. Tone was normal. Symmetrical absence of osteotendinous reflexes distally (styleradial, patellar, and Achilles). Bilateral plantar reflex in flexion.

Analytically, there was no leukocytosis (10.3 x 10^9^/L) or neutrophilia (3.7 x 10^9^/L). Hemoglobin was 14 g/dL, with no abnormalities in the electrolyte balance or renal function. Alkaline phosphatase was 526 IU/L, gamma-glutamyl transferase (GGT) was 1261.3 IU/L, and there was an increase in transaminases (ALT 157 IU/L and AST 68 IU/L). Total hyperbilirubinemia was 2.1 mg/dL, primarily due to direct bilirubin. The C-reactive protein (CRP) level was 0.56 mg/dL (Table 1).

**Table 1:** Analytical Progression Presented per Day of Hospitalization GGT: gamma-glutamyl transferase, INR: international normalised ratio, ALP: alkaline phosphatase, ALT: alanine transaminase, AST: aspartate aminotransferase, LDH: lactate dehydrogenase, CRP: C-reactive protein

Analytical Progression		D1	D3	D4	D5 (Date of Discharge)	Reference Values
White Blood Cells	x10^9^/L	10.30	6.50	5.60		4.5-11.5
Neutrophils	x10^9^/L	3.7	1.4	1.5		1.6-8
Lymphocytes	x10^9^/L	5.4	4.2	3.3		0.9-4
Hemoglobin	g/dL	14.0	13.6	13.3		12-15
Platelets	x10^9^/L	196	186	175		150-450
Prothrombin Time	s	10.9	11.0			
INR		0.92	0.93			
Urea	mg/dL	42	45			19-49
Creatinine	mg/dL	0.9	0.7			0.5-1.2
Sodium	mEq/L	136	135			135-145
Potassium	mEq/L	4.4	4.5			3.5-5
GGT	UI/L	1261.3	1158.6	1051.8	981.8	<38
ALP	UI/L	526	465	420	408	25-100
ALT	UI/L	157	151	151	160	3-31
AST	UI/L	68	84	88	99	3-31
LDH	UI/L		582	501		120-246
Total Bilirubin	mg/dL	2.1	1.5	1.4	1.30	0.2-1.1
Direct Bilirubin	mg/dL	1.54	1.05	0.96	0.9	0.1-0.5
CRP	mg/dL	0.56	0.25		1.30	<0.5
Folic Acid	ng/dL			6.3		1.0-20.0
Vitamin B12	pg/mL			920.0		179.0 – 1130.0

Due to distal areflexia, a lumbar puncture was performed, revealing cerebrospinal fluid (CSF) with a glucose concentration of 100 mg/dL, protein level of 70.8 mg/dL, and a mononuclear cell count of 14.67 mm^3^ per 1 mm^3^ of red blood cells. The infectious agent polymerase chain reaction (PCR) panel in the CSF was negative, and bacterial growth was absent; however, the panel did not include testing for EBV.

The cerebrospinal fluid examination was not consistent with the classic albuminocytological dissociation seen in Guillain-Barré Syndrome in the CSF. Instead, it was more suggestive of central nervous system infection or a parainfectious reaction. Serological tests for Brucellosis, typhoid fever, paratyphoid, and HIV were negative. Epstein-Barr virus serology revealed positive IgG and IgM antibodies (Table 2).

**Table 2:** Serology for Infectious Diseases EBC-VCA: Epstein-Barr Virus viral capsid antigen

Serology for Infectious Diseases		Date of Hospitalization	5 months after discharge	Reference Values
Wright Test				
Brucella abortus		Negative		--
Widal Test				
Antigen typhico H		Titer 1/320		--
Antigen typhico O		Negative		--
Antigen paratyphico B		Negative		--
Retrovirus Serology				
HIV C 1+2		Non-reactive		--
Epstein Barr				
Ac. VCA IgG	U/mL	116.00	205	<20 negative ≥20 positive
Ac. VCA IgM	U/mL	59.80	<10.00	≤20 negative 20-40 doubtful ≥40 positive
Syphilis Serology				
Treponema pallidum - total antibodies		Reactive	Non-reactive	--
Treponema pallidum IgM		Negative		--
RPR		Non-reactive		--

A repeat abdominal ultrasound did not show the previously mentioned alterations. The patient was diagnosed with polyradiculopathy in the context of Epstein-Barr virus infection and initiated intravenous immunoglobulin therapy. An electromyogram (EMG) was performed, indicating changes consistent with asymmetric, subacute (lasting more than 1 week) sensory-motor axonal polyneuropathy/polyradiculopathy, with greater involvement on the left side, suggesting an inflammatory/infectious etiology (Table 3).

**Table 3:** First electromyogram performed

Muscle	Interpretation	Spontaneus Activity					Voluntary Activity			
		Fib	PSW	Fasc	Amp	Dur	Poly	IP	Recruit	
Left Gastroc caput med	Neurogenic	1+	0		Normal	Normal	Normal	-	Reduced	
Right Gastroc caput med	Neurogenic	0	0		Normal	Normal	Normal	-	Reduced	
Left Rectus femoris	Central weakness	0	0		Normal	Normal	Normal	Low fq	Late	
Right Rectus femoris	Central weakness	0	0		Normal	Normal	Normal	Low fq l	Late	
Left Tensor fascia latae	Neurogenic	0	0		Normal	Normal	Normal	-	Reduced	
Right Tensor fascia latae	Neurogenic	0	0	1+	Normal	Normal	Normal	-	Reduced	
Left Tibialis anterior	Neurogenic	0	0		Normal	Normal	Normal	-	Reduced	
Right Tibialis anterior	Neurogenic	0	0		Normal	Normal	Normal	-	Reduced	

Clinical improvement was observed gradually from the third day of treatment. The patient completed a 5-day course of immunoglobulin therapy without complications during hospitalization. There was improvement in dysesthetic complaints in the lumbar and iliac region, with some lingering dysesthesias in the thigh that improved with movement and worsened with rest. The patient maintained a gait with a broad-based ataxia but with autonomy. Patellar osteotendinous reflexes were absent, while styleradial and Achilles reflexes were present (+), and bicipital and tricipital reflexes were more pronounced (++).

**Figure 1:**
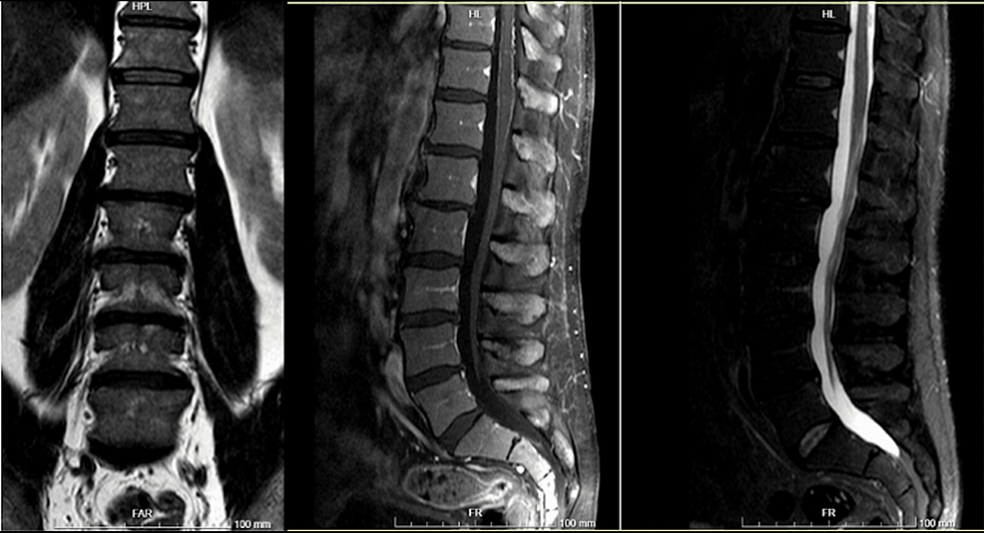
Lumbosacral magnetic resonance imaging (MRI) performed one week after the event

After being observed by physical medicine and rehabilitation, the patient was recommended for a rehabilitation program, but he declined outpatient treatment. A lumbosacral magnetic resonance imaging (MRI) performed one week after the event revealed preserved lumbar lordosis, misaligned posterior walls due to slight retrolysthesis of L1 over L2, degenerative changes in multiple lumbar segments, including arthrosic hypertrophy of posterior joints, hypertrophy of yellow ligaments, incipient osteophytosis, and circumferential disc bulges. These changes were accompanied by foraminal stenosis reduction at L3-L4 on the right and at L4-L5. Contrast-enhanced imaging showed mild intradural enhancement of roots (at L5-S1) and peri-medullary enhancement, likely venous in nature (Figure 1).

There was significant improvement in sensory and balance disturbances about one month after the event. The patient continued follow-up in the Internal Medicine and Neurology outpatient clinics. Anti-neuronal antibodies were requested but returned negative. A repeat electromyogram (EMG) one month after discharge was generally consistent with the previous findings, supporting the clinical hypothesis of polyradiculitis, bilateral post-ganglionic L5 involvement in a subacute phase, with mild severity on both sides but more pronounced on the right. Similarly, bilateral S1 involvement in a subacute phase with mild severity on both sides but more pronounced on the left (Table 4).

**Table 4:** Electromyogram one month after discharge

Muscle	Interpretation	Spontaneus Activity					Voluntary Activity			
		Fib	PSW	Fasc	Amp	Dur	Poly	Stabil	IP	Recruit
Left Gastroc caput med	Neurogenic	1+	1+	1+	+	+	+		-	Reduced
Right Gastroc caput med	Neurogenic	0	0	1+	+	+	+		-	Reduced
Left Rectus femoris	Normal	0	0		Normal	Normal	Normal	Normal	Normal	
Right Rectus femoris	Normal	0	0		Normal	Normal	Normal	Normal	Normal	
Right Tensor fascia latae	Neurogenic	0	0		+	+	+		-	Reduced
Left Tibialis anterior	Neurogenic	0	0	1+	+	+	+		-	Reduced
Right Tibialis anterior	Neurogenic	1+	1+		+	+	+		-	Reduced

A repeat EBV serology showed a strongly positive IgG (205 U/mL) and negative IgM (<10 U/mL), confirming seroconversion. Complete remission of neurological symptoms occurred six months after the acute event, and the patient remained asymptomatic to date.

## Discussion

Neurological manifestations of EBV infection can occur before, concurrently, or months after the primary EBV infection [3]. Published neurological disorders associated with EBV include diseases of the central nervous system, such as meningoencephalitis and transverse myelitis, as well as peripheral nervous system disorders, including demyelinating polyradiculoneuropathy, multiple mononeuropathy, multiple cranial neuropathies, brachial plexopathies, and lumbosacral radiculoplexitis [4].

The pathogenesis of these neurological alterations associated with EBV is not fully understood. It is believed that EBV activates antibody production by B lymphocytes, leading to the formation of immune complexes. Speculation about the mechanism of neuronal injury involves various theories: (1) infiltration of neuronal tissue by EBV-infected cells, which can directly affect neurons or cause a secondary inflammatory response; (2) induction of antibodies against neuronal tissue; or (3) an immune response to EBV that may cross-react with antigens in neuronal tissue [3,4]. The pathological response is not limited to the central nervous system, and concurrent or isolated involvement of peripheral nerves can occur [4-7]. Confirmation of primary Epstein-Barr virus infection can be established if seroconversion is demonstrated between acute and convalescent sera [8-10].

This case underscores the importance of considering viral etiologies, including EBV, in the differential diagnosis of acute polyradiculopathies, especially when atypical features are present. The clinical response to intravenous immunoglobulin therapy and the absence of relapse during follow-up support the efficacy of this treatment approach in EBV-related neurological manifestations. Further research is needed to elucidate the precise mechanisms linking EBV to polyradiculopathy and to optimize diagnostic strategies and therapeutic interventions for such cases.

## Conclusions

This case aims to draw attention to less frequent neurological complications that may occur in immunocompetent patients after primary EBV infection. This diagnostic possibility is not always considered, given its atypical nature, and there is a wide spectrum of neurological manifestations over an extended chronological period. Therefore, this entity should be suspected in cases presenting viral infection symptoms, as observed in the described patient, in combination with neurological symptoms such as polyradiculomyelitis, polyradiculitis, sudden cognitive disorders, meningitis, or meningoencephalitis. The inflammatory response is characterized by mononucleosis, often with atypical lymphocytes in the blood, in combination with pleocytosis and a decrease in the granulocyte-to-mononuclear leukocyte ratio in the cerebrospinal fluid.
